# Osteoclastogenesis Inhibitor and Antioxidant Properties of Konjac Glucomannan in a Periodontitis Mice Model: An In Vivo Study

**DOI:** 10.1155/2023/7400421

**Published:** 2023-10-31

**Authors:** Edlyn Dwiputri, Kartika Dhipta Lestari, Geraldi Hartono Kurniawan Tan, Benso Sulijaya, Yuniarti Soeroso, Sri Lelyati C. Masulili, Naoki Takahashi, Koichi Tabeta, Fatimah Maria Tadjoedin

**Affiliations:** ^1^Department of Periodontology, Faculty of Dentistry, Universitas Indonesia, Jakarta, Indonesia; ^2^Division of Periodontology, Graduate School of Medical and Dental Science, Niigata University, Niigata, Japan

## Abstract

**Background:**

Periodontitis is an inflammatory disease caused by specific microorganisms that gradually damage the periodontal and tooth-supporting tissues, thereby reducing a person's quality of life. Periodontal disease is closely associated with high reactive oxygen species (ROS) levels, with a high receptor activator of nuclear factor k*β* ligand (RANKL)/osteoprotegerin (OPG) ratio. Konjac glucomannan (KGM) is produced from the porang root, which has several properties. For example, it can reduce oxidative stress. The current study analyzed the osteoclastogenesis inhibitory and antioxidant properties of KGM based on histomorphometric findings, RANKL/OPG ratio, and ROS levels in the Swiss Webster mouse periodontitis model.

**Methods:**

Eight-week-old male Swiss Webster mice were divided into the nonligation, nonligation + KGM, ligation + *Porphyromonas gingivalis*, and ligation + *P. gingivalis* + KGM groups. KGM suspension was administered for 14 days. Periodontitis induction was performed from 7th to 14th day. On the 14th day, maxillae, gingival, and gingival crevicular fluid samples were collected to assess the histomorphometry of bone damage, gene expression ratio of RANKL/OPG, and ROS protein levels.

**Results:**

The periodontitis group pretreated with KGM presented with significantly reduced alveolar bone damage, RANKL/OPG ratio, and ROS level than without KGM group. KGM treatment had no harmful/toxic effects in mice.

**Conclusion:**

Administration of KGM could act as an adjunctive in periodontal therapy by suppressing periodontal disease via osteoclastogenesis inhibitory and antioxidant properties.

## 1. Introduction

Periodontitis is the leading cause of tooth loss in the adult population, and it can significantly affect the patients' quality of life [1, 2]. The 2019 Global Burden of Disease Study reported that periodontal diseases affected 20%–50% of the population worldwide, with increasing number of cases annually. Further, it was considered the second most important global oral disease burden after dental caries [3, 4]. Periodontitis is an inflammatory disease, which is associated with an increased risk of oxidative stress [5] in the oral cavity caused by dysbiosis of plaque biofilm, as well as the imbalance activity between the host inflammatory mediators and certain microbial bacteria. If oxidative stress is left untreated, oxidative damage can be observed based on the loss of gingival tissue, periodontal ligament, cementum, and alveolar bone [6, 7]. In addition, periodontal disease is strongly associated with systemic diseases, including diabetes mellitus, Alzheimer's disease, cancers, and atherosclerosis [7].

According to the European Federation of Periodontology S3 level clinical practice guidelines, subgingival biofilm and calculus can be eliminated with subgingival instrumentation and adjunctive therapy, such as physical or chemical agents, locally or systemically delivered host-modulating agents, and locally or systemically delivered antimicrobials [8]. However, adjunctive therapy such as systemic and local administration of antimicrobials has limitations in terms of ineffective doses and side effects. Moreover, the long-term use of antimicrobials can disrupt the oral microbiome ecosystem and develop antimicrobial resistance, which has become a major public health concern. Therefore, preventing periodontitis with alternative therapeutic biomaterials with fewer side effects has gained attention. For example, natural ingredients with bioactive compounds that have antioxidant properties are used because oxidative damage is commonly observed in periodontitis [5].

Konjac glucomannan (KGM) is a water-soluble polysaccharide isolated from the root of the porang plant *Amorphophallus konjac* [9–11]. KGM has antioxidant, antidiabetic, antiobesity, anti-inflammatory, anticancer, laxative, and prebiotic properties that can control human organs and organ systems [9–12]. KGM reduced the level of oxidative stress by increasing the level of enzymatic and nonenzymatic antioxidant by regulating the Nrf2 pathway [13]. Konjac oligosaccharides, a hydrolysis product from KGM, promoted bone health in calcium-deficient mice [14]. Moreover, KGM regulates the polarization of M1 to M2 macrophages [15, 16], which could prevent bone loss in murine periodontitis models [17]. Porang flour is commonly used in the food and health sector [18]. However, the role of KGM in regulating alveolar bone and its antioxidant property in periodontitis has not been explored. Therefore, the current study aimed to assess the osteoclastogenesis inhibitory and antioxidant properties of KGM by assessing the histomorphometry of bone damage, gene expression ratio of receptor activator of nuclear factor k*β* ligand (RANKL)/osteoprotegerin (OPG), and reactive oxygen species (ROS) protein levels in a Swiss Webster mice periodontitis model.

## 2. Materials and Methods

### 2.1. Reagents

KGM powder was obtained from Shimizu Chemical Corporation, Hiroshima-ken, Japan. Carboxymethyl cellulose (CMC) (Cipta-Kimia, Indonesia) was used to make a suspension.

### 2.2. Mice

Eight-week-old male Swiss Webster mice were obtained from the National Institute of Health Research and Development, Indonesian Ministry of Health (Jakarta, Indonesia). Mice were acclimated for 7 days prior to the procedures and were given food and drink ad libitum during the experimental period. The cages were cleaned twice a week to provide a clean environment. Picric acid (Sigma-Aldrich, St. Louis, MO, USA) was used to tag the mice based on the treatment groups. Forty-eight mice were randomly classified into the nonligation, nonligation + KGM, ligation + *Porphyromonas gingivalis*, and ligation + *P. gingivalis* + KGM groups. Each group comprised 12 mice. Mice were weighed daily using a digital scale until the 14th day to assess cytotoxicity. All research procedures were approved by the Ethics Committee of the Faculty of Medicine, Universitas Indonesia (No. KET-163/UN2.F1/ETIK/PPM.00.02/2022).

### 2.3. *Porphyromonas gingivalis* Culture

*P. gingivalis* W83 was cultured, as described in a previous study [19]. The number of colony-forming units was obtained via manual counting with the serial dilution technique.

### 2.4. KGM Treatment in Mice

To obtain the final concentration of 10 mg/mL, KGM suspension was synthesized by mixing KGM powder (Shimidzu, Japan) (80 mg/kg body weight) with 0.5% CMC suspension according to the dosage [13]. In the nonligation group, 2% CMC suspension was administered. Approximately 200 *µ*l/day of 80 mg/kg body weight of KGM or CMC suspension was administered daily for 14 days via oral gavage.

### 2.5. Induction of Periodontitis in Mice

**Figure 1:**
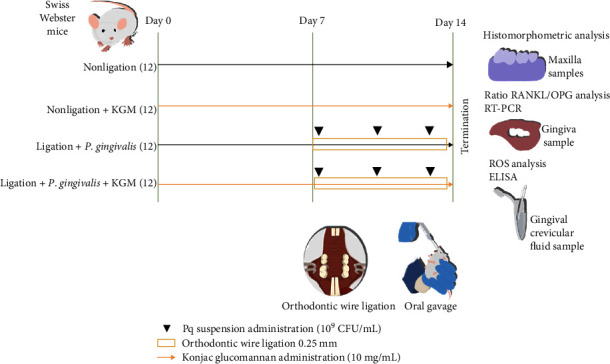
In vivo experimental design.

Periodontitis was induced by administering *P. gingivalis* suspension and wire ligature (Generic, China) on the 7th day. Mice were anesthetized with intraperitoneal injection of 10% ketamine (Ket-A 100, Peru) and 2% xylazine (Xyla, The Netherlands) (2 : 1) at a dose of 1.2 *µ*l/g body weight prior to wire ligature installation. A 0.25-mm wire ligature was cut into 3-mm long pieces, shaped into L-shape, and then inserted from the palatal side to the buccal side at the interproximal distance of the right maxillary first and second molars. The excess side of the ligature was bent to the shape/contour of the tooth to improve retention. *P. gingivalis* (10^9^ colony-forming units/mL) combined with 2% CMC at a dose of ∼200 *µ*l/day was administered to the ligation + *P. gingivalis* group via oral gavage every 3 days. The mice were euthanized under anesthesia with a cardiac puncture on day 14. Next, the maxillae, gingival tissues, and gingival crevicular fluid were collected.  Figure 1 shows the experimental design of this study.

### 2.6. Evaluation of Alveolar Bone Loss via Linear Histomorphometry

After removing the tissues and muscles attached to the bone, the maxillae samples were stored in 10% formalin solution and stained with 1% methylene blue (BioPharm, USA) to differentiate the cementoenamel junction line and the alveolar bone crest [20]. The root surface area of the distal maxillary first right molar and mesial maxillary second right molar was photographed with a macro camera (Nikon, Japan) and measured with the Image J software (National Institutes of Health, Bethesda, Maryland, USA). Histomorphometric measurements were performed by measuring the distance from the cementoenamel junction to the crest of the alveolar bone (mm). All measurements were performed in a blinded manner.

### 2.7. Assessment of Osteoclastogenesis Inhibitor Property

Gingival tissue samples were obtained from the right maxillary first and second right molar. Gingival sample collection was performed by making an incision with a scalpel. Then, the scalpel was gently withdrawn with a dental explorer. Samples were then stored in phosphate-buffered saline liquid and stored in −80°C freezer [1, 21]. The total RNA was extracted from the gingival tissue surrounding the ligated maxillary first and second molar using GeneZol (Geneaid, Taiwan), and then the levels of RANKL and OPG measured via a series of centrifugation through real-time polymerase chain reaction. A reverse transcriptase from the SensiFAST cDNA Synthesis Kit (Bioline, USA) was used to create a complementary DNA sequence from the RNA fragment. Complementary DNA samples were mixed with Master SensiFAST SYBR Hi-ROX (Bioline, USA) and primer and then analyzed using the Real-Time PCR System (Carl Zeiss, Jena, Germany). Total gene expression was calculated using the 2^−*∆∆Ct*^ method, which was compared to the glyceraldehyde-3-phosphate dehydrogenase value [22]. The primer (Integrated DNA Technologies, Singapore) sequences for the RANKL were as follows: forward, 5′-GGG TGT GTA CAA GAC CC-3′ and reverse, 5′-CAT GTG CCA CTG AGA ACC TTG AA-3′. The primer sequences for the OPG were as follows: forward, 5′-AGC AGG AGT GCA ACC GCA CC-3′ and reverse, 5′-TTC CAG CTT GCA CCA CGC CG-3′ [23]. The primer sequences for glyceraldehyde-3-phosphate dehydrogenase were as follows: forward, 5′-CAT CAC TGC CAC CA GAA GAC TG-3′ and reverse, 5′-ATG CCA GTG AGC TTC CCG TTC AG-3′ [24].

### 2.8. Investigation of Antioxidant Property or Reactive Oxygen Species Levels

Gingival crevicular fluid samples were obtained by placing paper points ISO 15 into the interproximal sulcus of palatal maxillary first and second right molars. Paper points were applied for 10 s before euthanasia was performed. Samples were stored in phosphate-buffered saline solution and stored in −80°C freezer [25]. ROS was measured using the enzyme-linked immunosorbent assay kits (BT lab, China). Optical density was measured at a wavelength of 450 nm using a microplate reader (Carl Zeiss, Jena, Germany).

### 2.9. Statistical Analysis

All data were presented as mean ± standard deviation. Statistical analyses were performed using GraphPad Prism version 9 (GraphPad Software, San Diego, California, USA). Data processing was started with the normality test using the Shapiro–Wilk test. Comparative linear histomorphometric findings, RANKL/OPG ratio, and ROS protein levels with a normal distribution between the two groups were analyzed using the *t*-test. *P*-values of <0.05 were considered as statistically significant.

## 3. Results

### 3.1. KGM-Related Cytotoxicity

**Figure 2:**
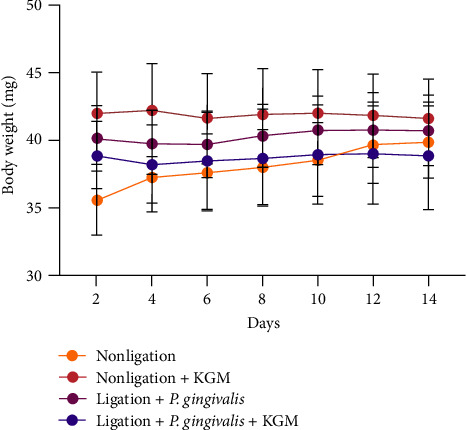
Body weight observation during the study. Data were presented as mean ± standard deviation in milligrams (mg) (*n* = 12 sample/group). ANOVA was used for comparisons between the groups.

All mice were considered healthy at the end of the experiment as there were no abnormalities or changes in body weight after KGM treatment during the experimental period (Figure 2). Therefore, KGM treatment had no toxic effects to the mice under administered of 14 days.

### 3.2. Alveolar Bone Destruction Suppression with KGM in a Periodontitis Mice Model

**Figure 3:**
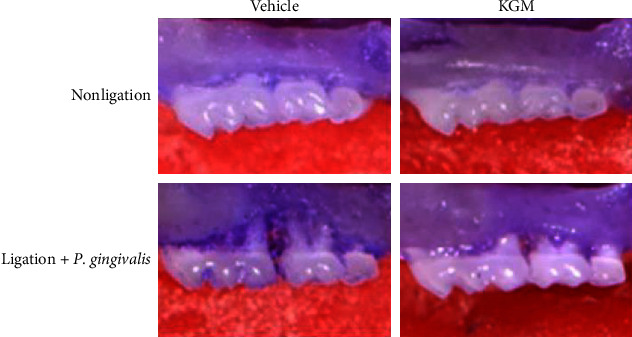
Effect of konjac glucomannan treatment on alveolar bone resorption, as shown on the histomorphometric images of the maxillae from each treatment group.

**Figure 4:**
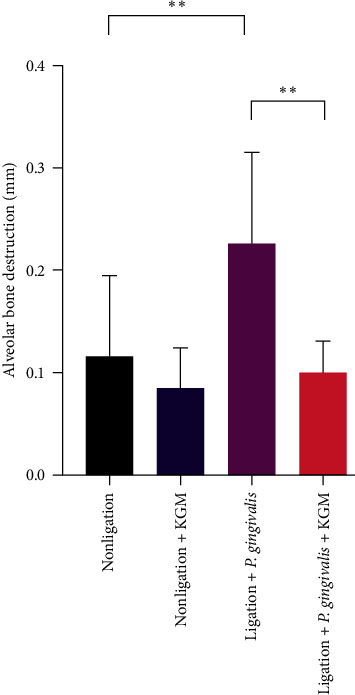
A quantification of alveolar bone loss after konjac glucomannan administration. Assessment was performed by measuring the distance from the cementoenamel junction to the crest of the alveolar bone (mm) on the root surface area of the distal maxillary first right molar and mesial maxillary second right molar (*n* = 3–6 samples/group). Data were presented as mean ± standard deviation. Significant values were indicated as  ^*∗∗*^*P* < 0.01.

Induction of an experimental periodontitis model in mice was performed with combined wire ligation and oral administration of *P. gingivalis* (Figure 1). The periodontitis-induced group had significantly greater alveolar bone destruction than the nonligated and KGM groups (Figures 3 and 4). These results confirmed the effects of the periodontitis induction technique in this in vivo study. KGM treatment alone did not show a difference in the appearance of the alveolar bone in the nonligated group. However, the mean value of alveolar bone destruction showed that the KGM group was lower than that of the nonligated group. Pretreatment with KGM significantly prevented alveolar bone loss, and the results were similar to those of the nonligated group (Figures 3 and 4). Therefore, KGM treatment inhibited alveolar bone destruction in our periodontitis model.

### 3.3. Osteoclastogenesis Inhibitory Effect of KGM

**Figure 5:**
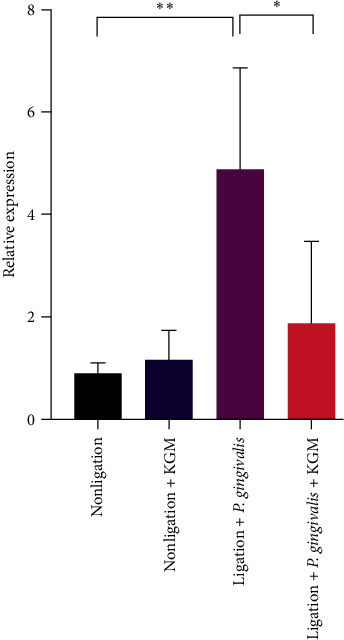
The gene expression ratio of receptor activator of nuclear factor k*β* ligand/osteoprotegerin in the gingiva was analyzed via real-time polymerase chain reaction. Data were presented as mean ± standard deviation (*n* = 6 samples/group). Significant values were expressed as  ^*∗∗*^*P* < 0.05 and  ^*∗∗*^*P* < 0.01. The *t*-test was used for comparisons between the two groups.

To investigate the possible mechanism involved in suppressing bone destruction by KGM, we examined the ratio of RANKL/OPG expression in the gingiva via real-time polymerase chain reaction. The periodontitis-induced group had a significantly higher gingival gene expression ratio of RANKL/OPG than the nonligated group. KGM treatment in the ligation + *P. gingivalis* group was more likely to decrease the gene expression ratio of RANKL/OPG in the gingiva compared with that in the periodontitis-induced group (Figure 5).

### 3.4. Antioxidant Effect of KGM

**Figure 6:**
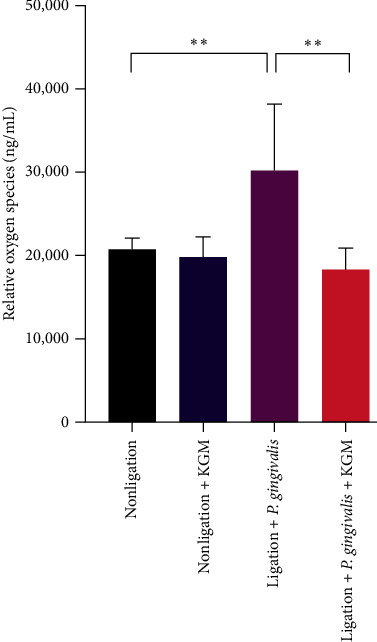
The reactive oxygen species protein level in the gingival crevicular sample was analyzed using enzyme-linked immunosorbent assay. Data were presented as mean ± standard deviation. Significant values were indicated as  ^*∗∗*^*P* < 0.01. The *t*-test was used for comparison between the two groups.

There was no significant difference in terms of the ROS levels in the nonligated groups with or without KGM administration. Surprisingly, we found a significant reduction in ROS in the gingival crevicular fluid of the periodontal disease-induced mouse group pretreated with KGM compared with the periodontal disease-induced mouse group (Figure 6). Hence, KGM treatment reduced the production of ROS in our periodontitis model.

## 4. Discussion

KGM is one of the most potential candidates for periodontitis prevention therapy as it shows antioxidant properties and can promote bone health, which become the main findings in our studies [13–15, 18, 26–29]. In the current study, we utilized 80 mg/kg body weight of KGM, which is an effective and nontoxic dose. Similar to a previous study, adverse side effects based on daily body weight were not observed (Figure 2) [13]. Moreover, the European Food Safety Authority (EFSA) proved KGM considered as nontoxic based on the results of acute oral toxicity studies for both human and animal with the limit of 10 g/kg per day [30]. However, the administration of 80 mg/kg body weight of KGM was found to be significantly effective against periodontitis (Figures 3–6).

Bone loss, which can be caused by increased oxidative stress, is observed in periodontitis [31]. To validate our mouse periodontitis model with ligation and *P. gingivalis* induction, we investigated bone alveolar loss histomorphologically. Results showed that the periodontitis group had greater bone loss than the control group. Hence, the effect of the periodontitis induction technique was validated in this in vivo study. Our results were similar to those of the study of Sulijaya et al. [19] That is, combined silk ligation and oral gavage of *P. gingivalis* caused significant bone destruction histomorphometrically within 7 days. This study used orthodontic wire ligation, in addition to *P. gingivalis* administration, in our mouse periodontitis model because it is an easier and more reproducible technique with greater alveolar bone damage than *P. gingivalis* and *P. gingivalis*-LPS treatments [32]. Results showed that KGM pretreatment significantly reduced alveolar bone reduction in the periodontitis group, which indicated the inhibition of alveolar bone damage by KGM. These findings were in accordance with those of Ai et al. [14] which showed that konjac oligosaccharides improved bone health based on bone mineral density, trabecular number, and cortical thickness by increasing calcium absorption and calcium retention rates in calcium-deficient mice [14]. Furthermore, several studies supported the results of this study that combined KGM with other ingredients. KGM powder and mulberry leave mixture led to cancellous bone mass and improved trabecular structure in mice [27]. In addition, KGM and k-carrageenan mixture caused increased proliferation of mouse bone marrow mesenchymal stem cells [28]. Bone mesenchymal stem cells in cell culture moved into the KGM nanofibrous scaffold [29].

We assessed the gene expression ratio of RANKL/OPG to understand the mechanism of bone loss repression to ameliorate periodontitis with KGM in a mouse periodontitis model. RANKL mediates osteoclastogenesis and causes bone resorption. Meanwhile, OPG inhibits bone breakdown and increases bone growth by inhibiting osteoclast activity. Further, the RANKL expression increased, whereas the OPG expression decreased in patients with periodontitis [25, 31]. Therefore, a high RANKL/OPG ratio is associated with osteoclastogenesis caused by excessive osteocytes apoptosis [7, 25, 31]. Our study showed that the periodontitis group had the highest increase in the gene expression ratio of RANKL/OPG. Pretreatment with KGM decreased the RANKL/OPG ratio in the periodontitis group, which indicated that KGM prevented alveolar bone resorption by inhibiting osteoclastogenesis in our mouse periodontitis model. This phenomenon might occur due to the role of macrophages. M1 macrophages, unlike M2 macrophages, increased the RANKL expression, which is associated with a high osteoclast activity and bone breakdown [33]. Meanwhile, KGM inhibited the production of proinflammatory cytokine and differentiation from M2 to M1 macrophages in the colon of mice with colitis [16] and in vitro [34]. Macrophages activated by KGM have an elongated shape, which is closely related to phenotype M2 macrophages, and can regulate the polarization of M1 to M2 macrophages [34]. However, future studies should be conducted to validate the underlying mechanism of osteoclastogenesis inhibition. Thus, the amelioration of periodontitis via KGM pretreatment was attributed to the ability of KGM in reducing ROS, which reduced alveolar bone loss and the RANKL/OPG ratio.

After identifying bone alveolar loss histomorphologically in a mouse periodontitis model and the cause of ROS-induced bone loss, the ROS levels were assessed. As expected, the ROS values were significantly higher in the gingival crevicular fluid. Periodontal disease is closely associated with excessive ROS levels [6]. ROS causes the release of proinflammatory cytokines and stimulates bone resorption, which plays an important role in the pathogenesis of periodontal diseases [35]. In the early phase of periodontitis, the number of neutrophils increases by stimulating *P. gingivalis*. Further, neutrophils arrive at the site of inflammation to eliminate the pathogen in response to the pathogenic biofilms [35]. Neutrophils can produce superoxide (O_2_^–^) via a metabolic pathway referred to as respiratory burst, which is catalyzed by NADPH oxidase during phagocytosis. Next, O_2_^–^ is released in the phagosomal and extracellular environment and converted into different radical and nonradical derivatives [6, 35]. Patients with periodontitis have a hyperreactive neutrophil phenotype, which increases the level of ROS [36]. With the increase of ROS levels, oxidative stress is triggered, and it causes oxidative damage to the periodontal tissue (alveolar bone, periodontal ligament, and gingival tissue). Thus, neutrophils are one of the sources of ROS in periodontitis [6]. We found that the gingival sulcus fluid of mice under periodontitis conditions had significantly higher ROS protein levels than that under control conditions (*P* < 0.05) (Figure 6). The oral administration of 80 mg/kg body weight of KGM had an antioxidant property by significantly reducing ROS in the periodontitis group pretreated with KGM compared with the periodontitis group that is untreated (*P* < 0.05), thereby reducing ROS under physiological condition. The reduction of ROS with KGM pretreatment may be attributed to the ability of KGM to enhance antioxidant enzymes (superoxide dismutase (SOD), catalase (CAT), and peroxidase (POD)) and nonenzymatic enzymes (vitamin C, glutathione (GSH)) [26]. Moreover, KGM was found to scavenge intracellular ROS in L929 cells via its H_2_O_2_ scavenging capability [15]. Moreover, pretreatment with KGM may enhance the expression of Keap-1, SOD, and HO-1 by modulating the Nrf2 signaling pathway [13]. HO-1 enhances run X2 level, which led to increased osteoblast differentiation. In addition, the nuclear factor of activated T cell and tartrate resistance acid phosphatase are inhibited and reduced in osteoclastogenesis [37].

The current study had several limitations. That is, the anti-inflammatory and antibacterial effects of KGM against *P. gingivalis* were not assessed. To better understand the efficacy of KGM against periodontitis, further studies must be conducted to assess these properties. Further, the drug delivery system of KGM should be investigated.

## 5. Conclusion

The current study first showed that KGM had osteoclastogenesis inhibitory and antioxidant properties by reducing alveolar bone destruction, gene expression ratio of RANKL/OPG, and ROS in our mouse periodontitis model. Thus, KGM can be a host modulation therapy for periodontal diseases in the future.

## Data Availability

The supporting data used to support the findings of this study are included within the article.
